# Analysis of *Nitraria Tangutourum* Bobr-Derived Fatty Acids with HPLC-FLD-Coupled Online Mass Spectrometry

**DOI:** 10.3390/molecules24213836

**Published:** 2019-10-24

**Authors:** Na Hu, Jian Ouyang, Qi Dong, Honglun Wang

**Affiliations:** 1 Key Laboratory of Tibetan Medicine Research, Northwest Institute of Plateau Biology, Chinese Academy of Sciences, Xining 810008, China; huna@nwipb.cas.cn (N.H.); qdong@nwipb.cas.cn (Q.D.); 2 Huzhou Plateau Biological Resource Centre of Innovation, Northwest Institute of Plateau Biology, Chinese Academy of Sciences, Huzhou 313000, China; ygzjj@126.com; 3 Qinghai Provincial Key Laboratory of Tibetan Medicine Research, Xining 810008, China

**Keywords:** fatty acids, *Nitraria tangutourum* Bobr, HPLC-FLD, online mass spectrometry, different geographical origins

## Abstract

Fatty acids (FAs) are basic components in plants. The pharmacological significance of FAs has attracted attentions of nutritionists and pharmaceutists. Sensitive and accurate detection of FAs is of great importance. In the present study, a pre-column derivatization and online mass spectrometry-based qualitative and quantitative analysis of FAs was developed. Nineteen main FAs were derivatized by 2-(7-methyl-1*H*-pyrazolo-[3,4-b]quinoline-1-yl)ethyl-4-methyl benzenesulfonate (NMP) and separated on reversed-phase Hypersil BDS C8 column with gradient elution. All FAs showed excellent linear responses with correlation coefficients more than 0.9996. The method obtained LOQs between 0.93 ng/mL and 5.64 ng/mL. FA derivatives were identified by both retention time and protonated molecular ion corresponding to *m*/*z* [M + H]^+^. A comparative study based on FA contents in peel and pulp, seeds and leaves of *Nitraria tangutourum* Bobr (NTB) from different geographical origins was performed with the established method. Results indicated that NTB were rich in FAs, and the types and contents of FAs varied among tissues. On the other hand, the same tissue of NTB from different geographical areas differed in the content, but not in type, of FAs.

Academic Editor: Daniel Cozzolino

## 1. Introduction

*Nitraria Tangutorum* Bobr. (NTB), belonging to the Zygophyllacea family, is one of the dominant and endemic species in Qinghai-Tibet Plateau of China [1,2]. NTB plays an important role in resistance to drought, high temperature, salinity and alkalinity and has been used as an ideal material for studying response and adaptability of plants to salinity stress [3]. The fruit of NTB is of high edible value and has been wildly used by locals in making juice, wine and tea. As a traditional herb and food, NTB fruit has been used to treat abnormal menstruation, heart diseases, neurasthenia and dyspepsia in west China [4,5]. Modern pharmacological studies showed that NTB fruit possesses multiple pharmacological activities such as hypoglycemia, hypolipidemia, antioxidation, antifatigue, immune regulation and protection against liver damage [5]. Moreover, NTB leaves were used to treat dizziness, headaches, stomach ailments and other digestive diseases [6]. Research showed that alkaloids extracted from NTB leaves exhibit significant anti-tumor activity [7]. Furthermore, NTB leaves were usually used for cattle and sheep feeding for its rich nutrients. The Qinghai-Tibet Plateau is abundant in NTB. Take the Qaidam Basin for example, where there is about 1000 km^2^ of NTB and the available fresh fruits are 1.5–2.5 × 10^5^ tons per year [8]. As a by-product in processing of NTB, NTB seed accounts for 35%–40% of the total mass and contains high level of fatty acids (FAs) [5,9]. Plenty of studies have indicated that FAs are indispensable for the pharmacological activities of medicinal plants [10,11]. FAs play a critical role in the prevention and treatment of coronary artery disease [12], cancer [13], diabetes [14], atherosclerosis [15], arthritis [16], hypertension [17] and other inflammatory and autoimmune disorders [18]. Thus, accurate determination of FAs is important for the quality control and safe use of NTB.

Accurate analysis of fatty acids with absorptiometry is challenging, because of its low content in organism and poor light absorption (both UV and visible light) and fluorescence response. The most usually used methods for FA analysis were GC or GC-MS, which have been applied to different research areas [19,20,21,22,23]. However, there existed a lot of shortcomings. For example, high temperatures are not suitable for thermally unstable FA derivatives, which usually make the data unsatisfactory [24]. In addition, explosiveness, toxicity and carcinogenicity of derivative reagents limit the usage of GC or GC-MS in FA analysis [25,26]. It has been proposed that the injection technique, especially vaporizing injectors, is one of the main sources of error in quantitative GC. Moreover, in ESI 450 °C often are required in order to active desolvation processes [26]. Compared with the GC method, HPLC-coupled with derivatization can overcome these disadvantages. For example, HPLC allows FA to be converted to a large number of different derivatives, and avoided the formation of less polar compounds as well as tailing peaks that significantly increased detection sensitivity [27]. Moreover, the availability of various strong UV-absorbing or fluorescent molecules, column packing materials and solvents can increase the selectivity and sensitivity of analysis by HPLC [28]. More importantly, low temperature during HPLC analysis can reduce the risk of isomerization of double bonds and the separated components can easily be collected and recovered from the mobile phase for further analysis with complementary techniques, such as mass spectrometry and nuclear magnetic resonance of infrared spectroscopy [29]. Therefore, pre-column derivatization combined with different fluorescence-labeling reagents has become the prevailing technique in FA analysis [30,31,32,33,34].

In the present study, a novel fluorescent reagent 2-(7-methyl-1*H*-pyrazolo-[3,4-b]quinoline-1-yl)ethyl-4-methyl benzenesulfonate (NMP) was used as the pre-column labeling reagent and was applied to the analysis of FAs in NTB. Considering the wide distribution of NTB in Qaidam Basin, we collected NTB samples from seven different geographical regions for the detection. To fully utilize the NTB resource, in addition to the previously studied NTB seeds, we also examined the FAs in peel, pulp and leaves of NTB. Generally, the aims of the present work were: (1) to develop a selective and sensitive method for the determination of FAs in *Nitraria tangutourum* Bobr of different origins by using NMP pre-column labeling and fluorescence detection which was coupled with online APCI-MS; (2) to compare the FA composition and content in NTB of different origins. Our study might provide a method for safety assessment and quality control in development of NTB-related products.

## 2. Results and Discussion

### 2.1. Optimization of Derivatization Conditions

#### 2.1.1. Effect of Co-Solvents on Derivatization

The presence of co-solvents directly affects efficiency of the derivatization reaction. In this study, DMF, acetonitrile, DMSO and THF were used as co-solvents, and their effects on the derivatization efficiency were investigated. The results showed that, with DMF as a co-solvent, the FA derivatives generated strong fluorescence and the derivatization efficiency was 1.5 to 2 times of that derived from other solvents-involved reactions. Meanwhile, due to the high solubility of lipids in DMF, the precipitation of hydrophobic long-chain FAs can be significantly reduced. Therefore, DMF was used as co-solvent in the following studies.

#### 2.1.2. Effect of Basic Catalysts on Derivatization

With DMF as co-solvent, the effects of different basic reagents on the catalytic effect of derivatization, including sodium carbonate, potassium oxalate, potassium carbonate and sodium acetate, were studied. Results showed that, compared with other catalysts, K_2_CO_3_ made the fluorescence intensity of the derivative product 1–2 times higher. Therefore, K_2_CO_3_ was used as the catalyst for the derivatization reaction. The highest derivatization reaction efficiency was obtained when the amount of K_2_CO_3_ was between 25 mg and 30 mg, and 25 mg was selected in following studies.

#### 2.1.3. Effect of NMP Concentration on Derivatization

With K_2_CO_3_ as a catalyst and DMF as a co-solvent, the influence of the amount of derivatizing reagent on the derivatization efficiency was investigated. Results showed that the derivatization reaction was insufficient when the amount of derivatization reagent was less than three times of that of the FAs. Along with the increase in amount of derivatization reagent, the fluorescence intensity increased. Fluorescence intensity reached the highest value when the amount of derivatizing reagent is five times of that of FAs, and no further increase of fluorescence intensity was observed when a higher amount of derivatizing reagents was added.

#### 2.1.4. Effect of Temperature and Time on Derivatization

Temperature was also an important factor affecting the efficiency of derivatization. When the temperature was lower than 80 °C, derivatization reaction was usually incomplete even after a long time. In contrast, when the temperature was set at 90 °C, the derivatization efficiency reached the highest. Interestingly, when the temperature increased further to 100 °C, the derivatization efficiency decreased. This may be due to the degradation of the derivatized product at high temperature. Ranging from 10 to 60 min at an interval of 10 min, the effect of reaction time on the derivatization efficiency was investigated. Results showed that the derivatization efficiency reached maximal value at 30 min.

**Figure 1:**
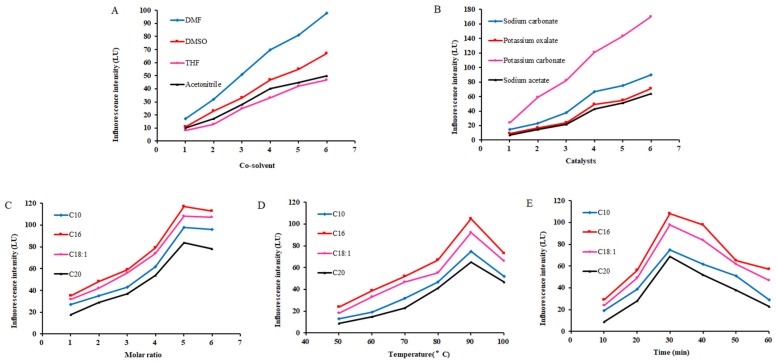
Optimization of derivatization conditions ((**A**): Co-solvent; (**B**): catalysts; (**C**): molar ratio; (**D**): temperature; (**E**): time).

In summary, our studies suggest the optimal derivatization conditions as follows: DMF as co-solvent, 25 mg of K_2_CO_3_ as catalyst, ratio between derivatizing reagent and FAs at 5:1, reaction temperature at 90 °C and reaction time for 30 min (Figure 1). Under these conditions, full reaction can be guaranteed.

### 2.2. Chromatographic Separation and Mass Spectrometry Identification

#### 2.2.1. HPLC Separation

In order to optimize the chromatographic separation conditions, the influence of the chromatographic column and mobile phase were investigated. Compared to C8 columns, C18 columns showed better separation effect for FAs. However, the separation time exceeded 100 min for C18 column-based separation. Therefore, C8 column was chosen for following experiments. Several columns such as Hypersil BDS C8 (200 mm × 4.6 mm, 5 μm), Zorbax Eclipse XDB-C8 (200 mm × 4.6 mm, 5 μm) and Hypersil GOLD C8 (200 mm × 4.6 mm, 5 μm) were tested and the separation efficiency was compared. Results showed that Hypersil BDS C8 column achieved complete separation of all 19 FAs within 52 min. For the mobile phase, usage of acetonitrile achieved faster separation and better resolution than methanol.

#### 2.2.2. MS Identification

**Figure 2:**
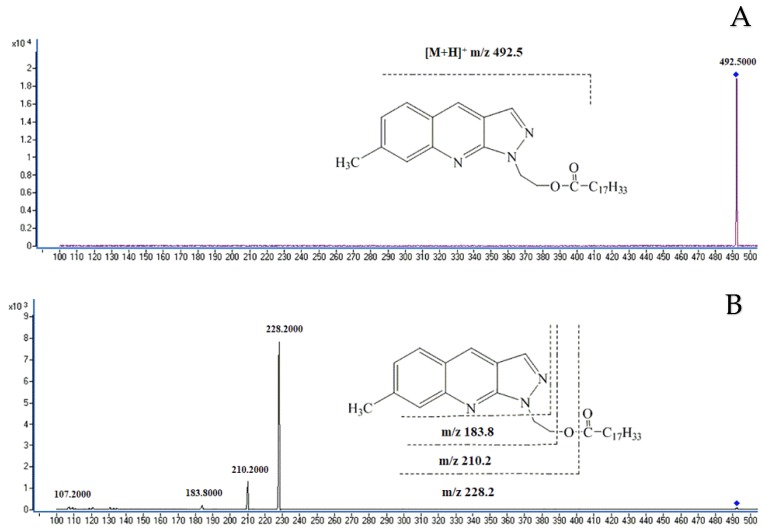
MS spectra of the representative oleic acid derivative and fragmentation pattern of the protonated molecular ion. (**A**) molecular ion peak of the oleic acid derivative. (**B**) fragmentation pattern of protonated molecular ion

The positive ion mode of the atmospheric pressure chemical ionization (APCI) was used to further identify the derivatives of FAs. The molecular ion peaks and fragment ion peaks produced by FA derivatives were listed in Table 1. Figure 2 showed the mass spectrum of oleic acid derivatization. *m*/*z* 492.5 was the molecular ion peak with high specific intensity. *m*/*z* 228.2, 210.2 and 183.8 are the fragment ion peaks generated after the molecular ion collision. The fragment ion peak *m*/*z* 228.2 was derived from cleavage of the O-CO bond of FA derivatives, while fragment ion peak 210.2 is from cleavage of the C-OCO bond from the FA derivatives. The fragment 183.8 was derived from the cleavage of N-C bond from the internal structure of the N-side chain. Fragment ion peaks *m*/*z* 210.2 and *m*/*z* 228.2 were valid indicator for existence of FA derivatives.

### 2.3. Method Validation

The established method was validated by linearity, limits of detection (LODs), limits of quantification (LOQs), precision and accuracy (Table 1). Linearity data was generated by plotting the peak areas versus corresponding concentrations of the 19 FA standards. The correlation coefficients were found to be > 0.996, which indicated excellent linearity of the analyses. LODs and LOQs are the concentration at which signal-to-noise ratio (S/N) is 3 and 10, respectively. The results showed that LODs and LOQs fell in the range of 0.43–2.03 ng/mL and 0.93–5.64 ng/mL, correspondingly. The precision was determined by parallel analysis of the actual sample three times. Results showed that precision of the method (expressed by relative standard deviation) is between 0.98% and 3.75%. To determine the accuracy, a recovery experiment was conducted and recovery rates between 91.8% and 103.2% for all FAs were obtained.

### 2.4. Analysis and Evaluation of FAs in NTB from Different Geographical Origins

**Figure 3:**
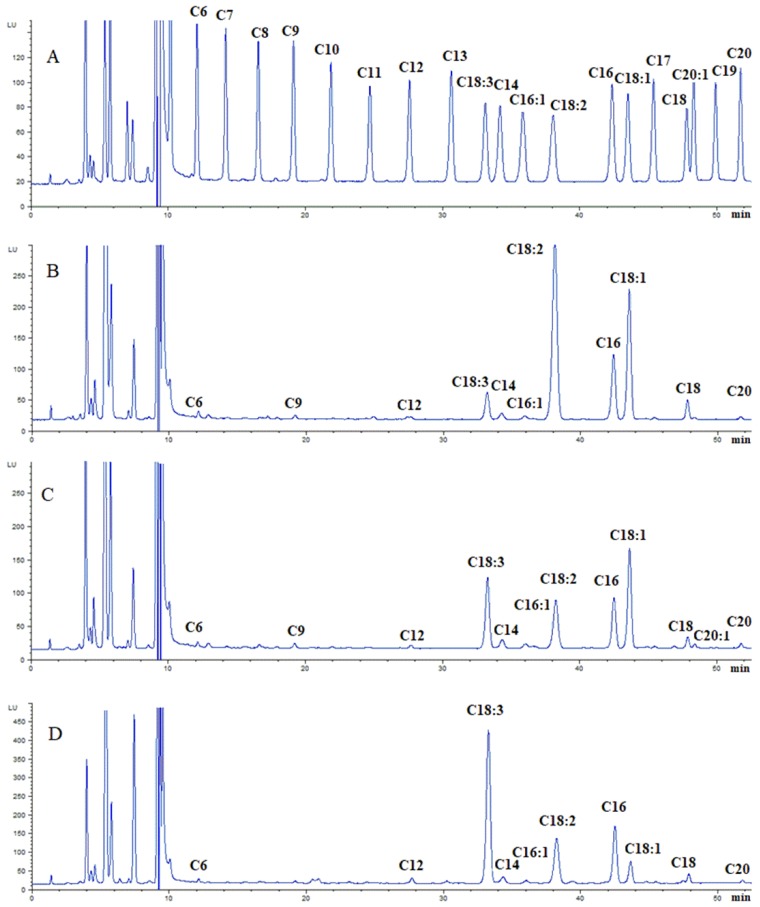
Representative chromatograms for standards (**A**), fatty acid derivatives in seeds of *Nitraria tangutorum* Bobr. (**B**), fatty acid derivatives in peel and pulp of *Nitraria tangutorum* Bobr. (**C**) and fatty acid derivatives in leaves of *Nitraria tangutorum* Bobr. (**D**).

The content of FAs in different tissues of NTB with different geographical origins was analyzed by the established method. The excess of the derivatizing reagent NMP could ensure full derivatization of all FAs in the samples. In addition, in order to ensure the accuracy of results, standard solution was measured after every five samples to compensate for the possible deviation in retention time. A representative chromatogram of FA standard solution and FAs in peel and pulp, seeds and leaves of NTB were shown in Figure 3. The detailed data of FA content in NTB was listed in Table 2, Table 3 and Table 4. 

Combined with Figure 3 and Table 2, Table 3 and Table 4, it can be seen that all tested tissues of NTB contain a large amount of unsaturated FAs, which accounted for more than 60% of total FAs. The content of unsaturated FAs in leaves was the highest, followed by seeds, peel and pulp. The main unsaturated FA varied among tissues. Analysis of FAs in NTB from different origins showed that unsaturated FA in the peel and pulp was mainly C18:1, with a content of 116.33–183.04 μg/g, followed by C18:3 (89.21–145.23 μg/g) and C18: 2 (72.43–125.16 μg/g). In addition, there existed a small amount of C20:1 (9.54–13.21 μg/g) and C16:1 (2.28–13.98 μg/g). In the Dulan area, the highest content of unsaturated FA was C18:2 (177.61 μg/g), followed by C18:1 (135.45 μg/g), C18:3 (112.11 μg/g) and C20:1 (12.31 μg/g), with C16:1 as the lowest content (3.06 μg/g). In the seed of NTB, the highest content of unsaturated FA was C18:2 (154.12–487.22 μg/g), followed by C18:1 (123.89–208.23 μg/g) and C18:3 (24.43–57.92 μg/g), with C16:1 as the lowest content (3.22–6.13 μg/g). The unsaturated FA in leaves of NTB was mainly C18:3 (566.24–1297.51 μg/g), followed by C18:2 (184.32–595.44 μg/g), C18:1 (39.56–163.29 μg/g) and C16:1 (9.29–21.21 μg/g). C20:1 was not detected in seeds or leaves of NTB. In terms of the saturated FAs, C16 and C18 were found to be the major constituents in different tissues of NTB. Their content in leaves was the highest, which was followed by seeds, and peel and pulp contained the lowest. The content of C16 in peel and pulp, seeds and leaves were 45.04–59.38 μg/g, 58.63–80.92 μg/g and 187.38–427.82 μg/g, respectively, while that of C18 were 23.01–56.51 μg/g, 31.67–88.90 μg/g and 48.24–89.05 μg/g, respectively. In addition to C16 and C18, a certain amount of C14, C20 and a small amount of C6, C9 and C12 was found in peel, pulp and seeds. Furthermore, a certain amount of C14 C20 and a small amount of C6, C12 was observed in leaves. In summary, there is basically no difference in the types of FAs in the same tissue of NTB from different areas, while the content could vary Peak labels: C6, hexanoic acid; C7, heptanoic acid; C8, caprylic acid; C9, pelargonic acid; C10, decoic acid; C11, undecanoic acid; C12, dodecanoic acid; C13, tridecanoic acid; C18:3, 8,11,14-octadecatrienoic acid; C14, myristic acid; C16:1, 9-hexadecenoic acid; C18:2, 9,12-octadecadienoic acid; C16, hexadecanoic acid; C18:1, 12-octadecenoic acid; C17, heptadecanoic acid; C18, octadecanoic acid; C20:1, 11-eicosenoic acid; C19, nonadecanoic acid; C20, arachidic acid.

## 3. Materials and Methods

### 3.1. Instruments

The HPLC analysis system was in an Agilent 1260 series HPLC (Agilent Technologies Co. Ltd., Palo Alto, CA, USA) equipped with a quaternary pump (model G1311C), an online-degasser (model G1322B), a thermostated column compartment (model G1316B), an autosampler (model G1329B) and an FLD detector (model G1321B). MSD Trap SL (model G2445D) from Bruker Daltonik (Bremen, Leipzig, Germany) was equipped with an atmospheric pressure chemical ionization (APCI) source (in positive ion model).

### 3.2. Reagents and Chemicals

All FA standards were purchased from Millipore Sigma (St Louis, MO, USA). HPLC grade acetonitrile and methanol were obtained from Yuwang Company, China. Dimethylformamide (DMF), dimethylsulfoxide (DMSO), tetrahydrofuran (THF), sodium carbonate (Na_2_CO_3_), potassium oxalate (C_2_H_2_K_2_O_5_), potassium carbonate (K_2_CO_3_) and sodium acetate (CH_3_COONa) of analytical grade were obtained from Shanghai Chemical Reagent Co. (Shanghai, China). All other reagents used were also of analytical grade unless otherwise stated. Pure water was from Wahaha Group Co., Ltd. (Hangzhou, China). The derivatization reagent NMP was synthesized in our laboratory [30].

### 3.3. Plant Material

Mature fruits and leaves of NTB in seven origins were collected from Qaidam Basin in October 2018 and were identified by senior engineer Changfan Zhou. The detailed sample information was listed in Table 5. The collected samples were dried naturally. Then, the seeds of fruits were separated from the peel and pulp. All dried sample were smashed and sieved through a 60 mesh sieve prior to analysis.

### 3.4. Preparation of Solutions

NMP solution (5.4 × 10^−3^ mol/L) was prepared by dissolving 20.47 mg NMP in 10 mL acetonitrile. Nineteen types of FA standard solution (2 × 10^−4^ mol/L) was obtained by diluting the corresponding stock solution (1 × 10^−2^ mol/L). All solutions were stored at 4 °C before HPLC analysis.

### 3.5. Preparation of Samples

200 mg tissue of *Nitraria tangutourum* Bobr (peel and pulp, seeds or leaves) was weighed into a 10 mL glass tube. 6 mL petroleum ether was added into the tube for ultrasonic extraction for 1 h. Then, the sample was centrifuged at 5000 r/min for 5 min and the supernatant was collected. After that, another 3 mL petroleum ether was added into the tube for second round of extraction. Supernatants of two extractions were combined and dried under a gentle nitrogen stream. Finally, the dried substance was dissolved in acetonitrile and exposed to HPLC analysis.

### 3.6. Derivatization Procedure

**Figure 4:**
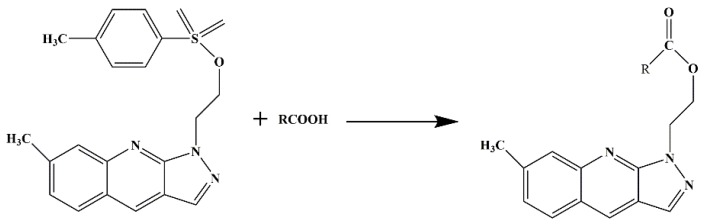
Derivatization reaction process of the derivatized reagent NMP and fatty acid.

20 µL of mixed FA standard, 25 mg K_2_CO_3_, 100 µl DMF and 200 µL NMP were added into a 2 mL vial. The vial was sealed and placed in 90 °C water bath for 30 min to make the derivatization complete. Then, the vial was taken out and cooled to room temperature. 250 µL of acetonitrile was added to dilute the reaction solution. The diluted solution was filtered through a 0.22 µm nylon filter and loaded directly to HPLC apparatus. The injected volume was set to 10 µL. The derivatization scheme of NMP with FAs is shown in Figure 4.

### 3.7. HPLC Separation and MS Condition

The mobile phases were A (100% acetonitrile) and B (5% acetonitrile and 95% water). The gradient condition was set as follows: 0–10 min, 40%–55%A; 10–25 min, 40%–75%A; 25–36 min, 70%–73%A; 36–42 min, 73%–83%A; 42–52 min, 83%–100%A. The temperature and flow rate of mobile phase were set to 35 °C and 1 mL/min. The column was equilibrated with the initial mobile phase for 8 min before the following analysis. Excitation and emission wavelength for fluorescence detection were at 245 nm and 410 nm, respectively. The chromatographic peaks were characterized by the retention time of the standard controls and further identified by online mass spectrometry. The mass spectrometer from Bruker Daltonik (model G2445D, Bremen Leipzig, Gremany) was equipped with atmospheric pressure chemical ionization (APCI) source (model G1947A). Ion source conditions were as follows: APCI in positive ion mode, nebulizer pressure 413 kPa, dry gas flow 5.0 L/min, dry gas temperature 350 °C, capillary voltage of 3500 V and corona current of 4 µA (Pos).

## 4. Conclusions

In this study, a method of simultaneous detection of saturated and unsaturated FAs with NMP labeling-based fluorescence detection and online MS analysis has been successfully established. The method was evaluated by LODs, LOQs, precision and accuracy, which showed good correlation and high sensitivity. The method was applied to analysis of FAs in the peel and pulp, seeds and leaves of NTB from different geographical regions. Results indicated that NTB were rich in FAs especially unsaturated FAs, and the types and contents of FAs varied among tissues. Meanwhile, the same tissues of NTB from different areas contain same kinds of FAs, although the content could differ.

## Figures and Tables

**Table 1:** MS data, linearity, correlation coefficient, limits of detection (LODs), limits of quantification (LOQs), precision and recovery for the quantification method of fatty acids.

Fatty Acid	MS [M + H]^+^	Linearity	Correlation Coefficient	LOD (ng/mL)	LOQ (ng/mL)	Recovery (%)	RSD (%)
C6	326.5	y = 5.86x + 8.21	0.9996	0.42	0.93	99.8	1.44
C7	340.5	y = 9.25x − 12.43	0.9998	0.54	1.24	96.5	2.56
C8	354.5	y = 6.21x + 1.45	0.9996	0.49	1.45	99.1	2.68
C9	368.5	y = 6.44x − 5.26	0.9997	0.57	1.57	97.5	3.14
C10	382.5	y = 4.86x − 8.24	0.9999	0.49	1.24	102.6	2.25
C11	396.5	y = 3.24x + 2.49	0.9996	0.62	1.65	98.3	0.98
C12	410.5	y = 6.72x + 0.24	0.9998	0.69	1.98	92.4	2.67
C13	424.5	y = 4.38x + 2.21	0.9997	0.78	2.14	99.5	3.21
C18:3	488.5	y = 7.25x + 4.46	1	0.86	2.35	98.9	3.65
C14	438.5	y = 6.24x + 7.98	0.9999	1.04	2.97	94.5	1.98
C16:1	464.5	y = 6.74x − 2.38	0.9998	1.15	3.01	91.8	2.56
C18:2	490.5	y = 8.99x - 5.66	0.9999	1.31	3.25	103.2	2.87
C16	466.5	y = 9.27x − 10.29	0.9996	1.42	3.96	97.6	1.99
C18:1	492.5	y = 8.02x + 6.08	0.9997	1.63	4.42	98.3	3.25
C17	480.5	y = 12.54 + 0.72	0.9997	1.71	4.54	95.5	2.76
C18	494.5	y = 4.36x + 2.58	0.9999	1.59	4.36	99.6	3.24
C20:1	520.5	y = 10.22x − 8.75	0.9996	1.87	5.23	92.7	2.96
C19	508.5	y = 12.45x + 14.57	0.9996	1.79	5.49	98.4	3.75
C20	522.5	y = 3.89x − 1.24	0.9998	2.03	5.64	99.5	3.29

**Table 2:** The measured contents of fatty acids in peel and pulp of *Nitraria tangutorum* Bobr. (μg/g).

Fatty Acid		Region					
	Dagele	Zongjia	Nuomuhong	Delingha	Hedong	Dulan	Gahai
C6	1.01 ± 0.03	1.15 ± 0.04	0.98 ± 0.03	0.87 ± 0.02	1.11 ± 0.04	0.94 ± 0.03	1.22 ± 0.05
C7	nd	nd	nd	nd	nd	nd	nd
C8	nd	nd	nd	nd	nd	nd	nd
C9	2.41 ± 0.08	3.07 ± 0.12	3.11 ± 0.11	2.09 ± 0.08	1.97 ± 0.07	2.06 ± 0.08	3.21 ± 1.21
C10	nd	nd	nd	nd	nd	nd	nd
C11	nd	nd	nd	nd	nd	nd	nd
C12	1.19 ± 0.05	1.43 ± 0.06	1.25 ± 0.05	1.07 ± 0.04	1.32 ± 0.04	1.65 ± 0.06	1.47 ± 0.05
C13	nd	nd	nd	nd	nd	nd	nd
C18:3	123.43 ± 4.94	90.21 ± 3.58	145.23 ± 5.81	89.21 ± 3.23	96.05 ± 3.62	112.11 ± 4.48	137.10 ± 5.41
C14	10.33 ± 0.43	12.02 ± 0.51	10.38 ± 0.40	10.40 ± 0.38	10.29 ± 0.42	15.41 ± 0.62	12.53 ± 0.53
C16:1	3.51 ± 0.15	13.98 ± 0.52	7.42 ± 0.27	3.68 ± 0.14	2.28 ± 0.06	3.06 ± 0.13	6.68 ± 0.18
C18:2	125.16 ± 5.00	155.62 ± 6.22	92.33 ± 3.63	84.47 ± 2.52	75.71 ± 3.02	177.61 ± 6.88	72.43 ± 2.44
C16	53.75 ± 2.15	57.65 ± 1.98	52.14 ± 2.02	45.04 ± 1.87	49.45 ± 2.00	59.38 ± 2.56	55.16 ± 2.46
C18:1	179.19 ± 7.12	139.98 ± 5.54	145.32 ± 5.60	181.82 ± 7.18	116.33 ± 4.65	135.45 ± 4.89	183.04 ± 6.78
C17	nd	nd	nd	nd	nd	nd	nd
C18	36.90 ± 1.34	39.43 ± 1.46	42.28 ± 1.50	42.04 ± 1.88	23.01 ± 0.94	56.51 ± 2.14	41.06 ± 1.78
C20:1	10.39 ± 0.42	13.21 ± 0.48	11.45 ± 0.44	9.54 ± 0.38	11.02 ± 0.44	12.31 ± 0.45	9.98 ± 0.36
C19	nd	nd	nd	nd	nd	nd	nd
C20	37.64 ± 1.45	50.22 ± 2.08	40.56 ± 1.64	41.41 ± 1.59	46.63 ± 1.82	63.48 ± 2.43	46.95 ± 2.25
Total Unsaturated Fatty Acids	441.68	413	401.75	368.72	301.39	440.54	409.23
Total Fatty Acids	646.77	651.17	634.45	592.19	523.56	716.08	627.56
Percentage of Unsaturated Fatty Acids	68.30%	63.42%	63.32%	62.26%	57.57%	61.52%	65.21%

**Table 3:** The measured contents of fatty acids in seeds of *Nitraria tangutorum* Bobr. (μg/g).

Fatty Acid		Region					
	Dagele	Zongjia	Nuomuhong	Delingha	Hedong	Dulan	Gahai
C6	0.67 ± 0.02	0.74 ± 0.02	0.53 ± 0.01	0.62 ± 0.02	0.98 ± 0.03	0.54 ± 0.01	0.76 ± 0.02
C7	nd	nd	nd	nd	nd	nd	nd
C8	nd	nd	nd	nd	nd	nd	nd
C9	1.12 ± 0.04	1.31 ± 0.05	0.98 ± 0.04	1.21 ± 0.05	0.87 ± 0.03	1.15 ± 0.04	1.22 ± 0.05
C10	nd	nd	nd	nd	nd	nd	nd
C11	nd	nd	nd	nd	nd	nd	nd
C12	2.10 ± 0.07	2.42 ± 0.08	2.51 ± 0.09	3.20 ± 0.11	2.15 ± 0.09	2.33 ± 0.08	1.85 ± 0.07
C13	nd	nd	nd	nd	nd	nd	nd
C18:3	24.43 ± 0.92	28.75 ± 0.98	39.26 ± 1.34	52.38 ± 2.08	51.73 ± 1.99	57.92 ± 2.28	46.31 ± 1.86
C14	5.23 ± 0.21	6.56 ± 0.25	6.31 ± 0.24	9.34 ± 0.33	8.48 ± 0.30	11.36 ± 0.42	10.78 ± 0.38
C16:1	3.22 ± 0.13	4.51 ± 1.13	4.38 ± 0.14	3.56 ± 0.15	5.76 ± 0.18	4.02 ± 0.15	6.13 ± 0.22
C18:2	219.33 ± 8.56	487.22 ± 14.23	234.65 ± 7.88	215.18 ± 7.14	154.12 ± 5.89	232.15 ± 6.84	256.38 ± 7.23
C16	67.66 ± 2.32	80.92 ± 2.98	78.45 ± 3.09	59.57 ± 2.10	58.63 ± 2.56	76.00 ± 2.68	63.43 ± 2.51
C18:1	128.98 ± 5.14	208.23 ± 7.88	130.57 ± 4.08	125.34 ± 3.96	123.89 ± 4.02	127.62 ± 3.80	138.90 ± 5.02
C17	nd	nd	nd	nd	nd	nd	nd
C18	31.67 ± 1.16	87.85 ± 3.04	74.65 ± 3.11	88.90 ± 2.98	42.02 ± 1.43	72.33 ± 2.56	69.42 ± 2.32
C20:1	nd	nd	nd	nd	nd	nd	nd
C19	nd	nd	nd	nd	nd	nd	nd
C20	23.22 ± 0.68	36.54 ± 1.28	28.90 ± 0.60	34.86 ± 1.11	41.09 ± 1.23	29.82 ± 0.98	38.75 ± 1.34
Total Unsaturated Fatty Acid	375.96	728.71	408.86	468.72	335.5	421.71	447.72
Total Fatty Acids	525.48	964.47	617.90	614.37	480.42	642.27	656.18
Percentage of Unsaturated Fatty Acids	71.55%	75.56%	66.17%	76.30%	69.83%	65.66%	68.23%

**Table 4:** The measured contents of fatty acids in leaves of *Nitraria tangutorum* Bobr. (μg/g).

Fatty Acid		Region					
	Dagele	Zongjia	Nuomuhong	Delingha	Hedong	Dulan	Gahai
C6	1.23 ± 0.02	0.98 ± 0.01	1.52 ± 0.05	0.96 ± 0.03	1.44 ± 0.03	0.58 ± 0.01	1.76 ± 0.06
C7	nd	nd	nd	nd	nd	nd	nd
C8	nd	nd	nd	nd	nd	nd	nd
C9	nd	nd	nd	nd	nd	nd	nd
C10	nd	nd	nd	nd	nd	nd	nd
C11	nd	nd	nd	nd	nd	nd	nd
C12	4.03 ± 0.14	4.53 ± 0.13	5.12 ± 0.21	3.02 ± 0.11	2.77 ± 0.09	3.24 ± 0.12	3.34 ± 0.08
C13	nd	nd	nd	nd	nd	nd	nd
C18:3	870.96 ± 30.4	566.24 ± 21.2	657.43 ± 23.6	1297.51 ± 48.4	910.2 ± 32.8	726.51 ± 28.0	802.41 ± 32.6
C14	24.20 ± 0.76	25.57 ± 0.78	22.51 ± 0.65	22.51 ± 0.72	19.95 ± 0.69	19.65 ± 0.74	21.38 ± 0.83
C16:1	20.30 ± 0.76	16.78 ± 0.58	13.45 ± 0.42	9.29 ± 0.81	17.56 ± 0.64	12.57 ± 0.43	21.21 ± 0.65
C18:2	208.12 ± 8.02	184.32 ± 6.78	211.23 ± 7.11	595.44 ± 16.89	186.71 ± 7.02	364.46 ± 10.65	324.88 ± 11.63
C16	232.42 ± 8.88	187.38 ± 7.04	244.45 ± 9.02	427.82 ± 12.87	210.80 ± 7.69	304.17 ± 11.28	268.04 ± 8.06
C18:1	123.24 ± 3.87	129.51 ± 4.04	133.86 ± 3.98	57.31 ± 2.02	39.56 ± 1.56	163.29 ± 5.60	102.53 ± 3.66
C17	nd	nd	nd	nd	nd	nd	nd
C18	57.82 ± 2.10	53.86 ± 2.02	48.92 ± 1.98	68.68 ± 2.37	48.24 ± 1.96	89.05 ± 3.19	52.42 ± 2.01
C20:1	nd	nd	nd	nd	nd	nd	nd
C19	nd	nd	nd	nd	nd	nd	nd
C20	53.71 ± 2.12	47.07 ± 1.67	45.65 ± 1.56	80.25 ± 2.98	71.38 ± 2.78	81.86 ± 3.10	69.55 ± 2.52
Total Unsaturated Fatty Acid	1260.7	932.32	1041.95	1990.42	1195.86	1305.91	1285.55
Total Fatty Acids	1669.43	1265.94	1434.86	2688.12	1587.85	1875.41	1757.83
Percentage of Unsaturated Fatty Acids	75.52%	73.65%	72.62%	74.05%	75.31%	69.63%	73.13%

**Table 5:** Information of the collected *Nitraria tangutorum* Bobr. sample in the Qaidam Basin.

Origin	Elevation (m)	Longitude	Latitude
Dagele	2679	95°45.202′	36°27.216′
Zongjia	2778	96°56.850′	36°15.959′
Nuomuhong	2703	96°28.233′	36°32.338′
Keluke Lake	2816	96°54.180′	37°19.024′
Hedong Farm	2783	96°07.799′	36°25.657′
Dulan	3198	97°59.375′	36°01.921′
Gahai	2854	97°35.766′	37°07.535′
